# Perceived Expressed Emotion, Emotional and Behavioral Problems and Self-Esteem in Obese Adolescents: A Case-Control Study

**DOI:** 10.4274/jcrpe.0101

**Published:** 2018-11-29

**Authors:** Merve Çolpan, Şafak Eray, Erdal Eren, Ayşe Pınar Vural

**Affiliations:** 1 Çanakkale State Hospital, Clinic of Child and Adolescent Psychiatry, Çanakkale, Turkey; 2 Uludağ University Faculty of Medicine, Department of Child and Adolescent Psychiatry, Bursa, Turkey; 3 Uludağ University Faculty of Medicine, Department of Pediatric Endocrinology, Bursa, Turkey

**Keywords:** Obesity, self-esteem, expressed emotion, psychopathology

## Abstract

**Objective:**

Obesity is a chronic disease which leads to medical and psychiatric complications. Family climate is a critical factor in the treatment of obesity and comorbid psychiatric disorders. In our study, perceived expressed emotion (EE), psychopathology, self-esteem and emotional and behavioural problems (BP) among obese adolescents were investigated and compared with their non-obese peers.

**Methods:**

The subjects were 49 obese adolescents and 47 non-obese adolescents served as the control group. All participants were requested to fill out the Socio-demographic Data Form, Shortened Level of Expressed Emotion Scale, Rosenberg Self-Esteem Scale, Strength and Difficulties Questionnaire-Adolescent Form.

**Results:**

In our study, obese adolescents showed a significant difference in perceived EE (p<0.001). Subscales of EE, such as Lack of Emotional Support (p<0.001), intrusiveness (p<0.001), irritability (p<0.001), self-esteem (p<0.001), emotional and BP (p<0.001), attention deficit-hyperactivity disorder (p<0.001), problems in peer relationships (p<0.001) and social skills (p<0.001) were significantly worse when compared with the control group. There was a strong relationship between EE and emotional and BP and self-esteem.

**Conclusion:**

The higher rate of perceived EE, psychopathology and low self-esteem among obese adolescents showed that obesity prevention and treatment are also crucial for good mental health in adolescents. The important role of the family in mental health of obese adolescents was emphasized. It was shown that identification of risk factors in childhood that promote obesity should be done so that targeted intervention and prevention programs can be developed.

**What is already known on this topic?**

Family climate is important for the mental health of youths. Expressed emotion (EE) is a measure of the family climate. Studies have shown that family climate and thus EE is important in prevention and treatment of obesity. Although family attitude and psychopathology is an important issue in obesity treatment, there is only one published study on children and one on adults in this field.

**What this study adds?**

In this study, the relationship between perceived expressed emotion (EE) and emotional and behavioural problems (BP) and self-esteem in obese adolescents was investigated. A higher rate of perceived EE, emotional and BP were observed in the obese group than in the control group. According to our findings, the obese adolescents had significantly lower levels of self-esteem than the control group. To the best of our knowledge, no study concerning EE and psychopathology and self-esteem of obese adolescents has been conducted in Turkey.

## Introduction

The prevalence of obesity among young people has recently increased dramatically in both developed and developing countries (1). Obesity affects all physiological systems of humans at every age. Obese adolescents exhibit psychiatric problems such as body dissatisfaction, lower self-esteem, depression and attention deficit-hyperactivity disorder (ADHD) (2,3,4). Behavioral problems, neurocognitive function deficits and ADHD symptoms in childhood have been found to affect weight gain over time (5). Pathological family environment factors, such as mental illness and inadequate care, are also found to be strongly associated with the psychopathology of obesity in children (5). In this context, family-based programs have been shown to be effective in targeting childhood obesity (6).

Expressed emotion (EE) is a measure of the family climate in the home which is characterized by communication styles of family members, such as emotional support, irritability and intrusiveness. This concept was formulated because of the strong relationship between environmental changes in the family system and the mental health of family members (7). In considering the association between obesity and mental health, EE becomes an important factor. Although the relationship between EE and mental-physical illnesses is well understood, researchers generally focus on the relationship between EE and eating disorders such as anorexia nervosa, bulimia, binge eating and emotional eating (8,9).

However, EE is an under-investigated issue in obesity. While the importance of the effect of the family on obesity has been shown (10,11), there is still little information regarding the difference between perceptions of EE of obese adolescents and those of their healthy peers. To the best of our knowledge, only one study regarding EE and obesity in children has been conducted to date (7). These researchers concluded that childhood obesity intervention programs may benefit from targeting maternal psychopathology, EE and coping skills. Also there is only one poster report of a study in the adult literature regarding obesity and EE (12). This study concluded that levels of EE should be considered when planning treatment interventions to enhance compliance in obese patients.

The goal of the current study was to investigate the perception of the family climate, as well as the emotional and behavioral problems of obese adolescents, by comparing obese adolescents with a non-obese control group. A second objective was to examine the association between perceived EE and psychopathology and self-esteem in obese adolescents. We also aimed to investigate the role of the family in the mental health of obese adolescents, as well as in their treatment and to identify the risk factors in childhood that promote obesity. Identifying these risk factors will help to develop targeted intervention and prevention programs.

## Methods

Subjects and controls were recruited at the Paediatric Endocrinology Outpatient Clinic of Uludağ University Medical School between January 1 and July 31, 2015. The control group of adolescents was matched to the patient group for age and gender. The necessary legal permission and approval were obtained from the Uludağ University Ethics Committee (Date: 14.11.2014, No: 2014-19/5) before proceeding to the data collection stage. All participants in the study and their parents gave informed consent after being informed of the methods and objectives of this study. A child and adolescent psychiatrist evaluated all participants using a clinical interview, based on Diagnostic and Statistical Manual of Mental Disorders (13). The exclusion criteria were the presence of a neurological disorder, clinical mental retardation, autism spectrum disorder, physical disability with significant functional impairment, alcohol and/or substance abuse and any clinical conditions that cause obesity, such as steroid use or antipsychotic drug use. The weight and heights of adolescents were measured by the same nurse working in our outpatient clinic and body mass index (BMI) was calculated as weight in kilograms divided by height in square meters. BMI-standard deviation (SD) score (SDS) and BMI percentiles were calculated using age and gender-specific norms published by the Centers for Disease Control and Prevention (14). To compare BMI across different ages, BMI Z-scores were also calculated. The Z-score represents the number of SD above or below the considered population mean value based on standardized tables for children. Obesity was defined as a BMI Z-score value above 2 SD for age and gender. Adolescents who had a BMI above the 95^th^ percentile for age and gender or a BMI SDS above +2.0 SD were defined as obese and taken into the study group (15). All participants were requested to fill out a Strength and Difficulties Questionnaire (SDQ)-Adolescent Form and the socio-demographic form prepared by the researchers. EE was assessed using the Shortened Level of Expressed Emotion Scale (S-LEES), the Rosenberg Self-Esteem Scale (RSES) assessed self-esteem and SDQ.

### Data Collection Materials

### Socio-demographic Form

The “Socio-demographic Form” was created by the researchers. Data items collected included age, gender, educational level of the parents, employment status of the parents, number of siblings, birth order and economic status. Income levels were determined based on the official starvation and poverty limits of 2015.

### S-LEES in Adolescents

This was developed by Nelis et al (16) and was translated into Turkish by Vural et al (17) in 2013. S-LEES consists of 33 items measuring the EE of the person perceived to be most important in the participant’s life over the previous three months. The three subscales of the S-LEES include Lack of Emotional Support (LES), irritability and intrusiveness. Higher scores indicate higher levels of EE. Nelis et al (16) reported Cronbach’s alpha coefficients, a measure of reliability, of 0.88, 0.82 and 0.70 for the S-LEES, irritability and intrusiveness subscales, respectively. When the reliability scale was evaluated by Vural et al (17), Cronbach’s alpha coefficient was found to be as high as 0.90. The forms were completed by the adolescents themselves. Items were answered on a Likert scale with four units ranging from “1 (not true)” to “4 (true)”.

### SDQ - Adolescent Form

SDQ was translated into Turkish by Güvenir et al (18). It was developed in 1997 by British psychiatrist Goodman (19) for screening behavioral and emotional problems in children and adolescents. The SDQ has 25 items evaluating both positive and negative emotions and behavioral features. These items are grouped into five sub-scales, each containing five items, according to both appropriate diagnostic measures and the results of factor analysis: ADHD, behavioral problems, emotional problems, peer relationship problems and social behaviors. In Turkey, the Cronbach’s alpha for the SDQ was reported to be 0.73 by Güvenir et al (18).

### RSES

The RSES was adopted to evaluate self-esteem in children and adolescents (20). This scale consists of 12 sub-tests, with the first ten articles aiming to evaluate self-esteem. The numerical levels of self-esteem are evaluated as: 0 to 1 points - high, 2 to 4 points- medium and 5 to 6 points- low. In our study, we used the first subtest to evaluate self-esteem. In Turkey, the validity and reliability of the scale was tested by Çuhadaroğlu (21).

### Statistical Analysis

Data were evaluated using IBM Statistical Package for the Social Sciences Statistics version 22 program (22). The median and range were used for descriptive statistics of the groups. Comparison of the non-normally distributed variables and non-parametric parameters was performed using the Mann-Whitney U test. Comparison of the categorical variables of the groups was performed using the chi-square tests. The majority of data were not normally distributed. Therefore, data were analyzed using nonparametric analysis. The correlations were performed using Spearman’s rho testing. A p-value of <0.05 was considered statistically significant.

## Results

A total of 108 participants were initially recruited. However 12 subjects were excluded for the following reasons: two subjects with clinical intellectual disabilities, two with epilepsy, two who had had treatment with exogenous steroids and six who did not complete all of the questionnaires. Thus the study was conducted on 49 obese subjects and 47 healthy adolescents formed the control group.

The overall median age was 14 years. The median age of the obese group was 14 (minimum: 12-maximum: 17). 52.0% (n=26) of the obese group were girls. The median age of the control group was 15 (minimum: 12-maximum: 17) and 56.0% (n=28) were girls. The groups were similar in terms of age (p=0.175) and gender (p=0.841).

To compare socio-economic status (SES), the poverty line defined as an income of 250 dollars/month and the hunger line of 400 dollars/month, as defined by the Turkish Trade Union Confederation, were used (23). The groups were divided into high and low SES subgroups. In terms of family income, the difference was not statistically significant between the patient and control groups. The groups were also similar in terms of cohabitation of parents, mothers’ and fathers’ educational levels and living conditions (p>0.05).

The perceived EE scores and the S-LEES scores of the patient and control groups are shown in Tables 1 and 2. There was a significant difference in the total perceived EE scores and also a significant difference in the subscales concerning irritability and intrusiveness between the two groups.

All cases were evaluated using the RSES. There was a statistically significant difference between the obese and control groups (p<0.001) (Table 1).

The presence of psychopathology in the two groups was evaluated using SDQ. There was a statistically significant difference between the obese group and the control group in the subscales of SDQ as follows: “emotional problems” (p<0.001), “behavioral problems” (p=0.001), “attention deficiency and hyperactivity” (p<0.001) and “peer relationship problems” (p<0.001). However, there was no significant difference in prosocial behaviors when comparing the two groups (p=0.077) (Table 2).

Correlations between EE scores and RSES scores and SDQ scores in the obese group are shown in Table 3. A strong correlation was found between EE emotional problems (p=0.012, r=0.36), behavioural problems (BP) (p=0.107, r=0.23), and self-esteem (p<0.001, r=0.56) in the obese group (Table 3).

## Discussion

Although obesity is a common disorder, its social determinants and psychosocial consequences are still inadequately understood. Our results emphasize that family climate, as evaluated by EE in obese patients, is essential. Prevention and treatment of obesity are also necessary for the mental well-being of these adolescents. When the vulnerability and tendency to psychiatric disorders of obese adolescents is considered, it can be seen that there is a need for further studies regarding obese adolescents and their families in regard of the psycho-social interactions which occur in families. The findings of our study, showing significant differences in the intra-family psychosocial interactions when comparing obese and non-obese adolescents, suggests that this aspect merits further investigation and may help to improve management of interventions in the future.

In this study, we investigated perceived EE, psychopathology and self-esteem of obese youths by comparing them with their non-obese peers. A higher rate of perceived EE was observed in the obese group than in the control group. Also a higher rate of emotional and BP were observed in obese adolescents. According to our findings, the obese adolescents had significantly lower levels of self-esteem than the control group. Similarity of the study and control groups in terms of age and gender strengthens the validity of our results.

Studies have been conducted on interactions in the families of obese children (24). The families of obese children have been reported to be more angry and critical towards to their obese children (10). In studies of family functioning and obesity, it has been reported that families of obese children are more dysfunctional than those of their non-obese peers (11). In addition, inappropriate parental attitudes were found to be associated with increased risk for abnormal eating behaviour and obesity (25). EE is an empirical concept that was developed for evaluating family climate. When we examined the perceptions of adolescents regarding their families’ EE, the obese adolescents perceived significantly lower levels of emotional support, higher irritability and greater intrusiveness. The studies investigating the parents of obese adolescents and EE also recommended taking EE into account while providing treatment for obesity (12).

In the literature dealing with obesity and psychopathology a strong association between emotional and BP, peer problems and obesity has been shown (26,27,28,29). A strong relationship between ADHD and obesity was also reported which is in keeping with our findings. There are studies underlining the role of abnormal eating pattern, possible genetic factors and sedentary life style of patients with ADHD and obesity (26,29). There are also studies reporting a causal role of ADHD in contributing to weight gain (29). According to a longitudinal study, anxiety and depression are more common in children and adolescents with obesity (27). Our results again support these findings in adolescents.

Self-esteem in obese adolescents was another issue investigated in our study. Lower self-esteem in obese adolescents has been reported in several studies (2,30,31). A strong relationship between body shame and vulnerability to eating problems has been reported and this increases the risk of low self-esteem and eating disorders among both obese and non-obese youngsters (32). A 4-year follow-up study by Strauss (30) with 1520 participants found that self-esteem was significantly lower in obese adolescents compared to non-obese adolescents, again in line with our findings. Our results are also consistent with previously published reports which show a higher ratio of psychiatric problems, such as depression, BP and low esteem, among obese adolescents when compared to non-obese adolescents.

In our study a strong association was observed between EE and emotional behavioral problems and self-esteem of obese adolescents. This has been previously reported. When we investigate the subscales of EE, the relationship between LES seems to be critical in the mental health of obese adolescents. Adolescents who perceived their parents as less emotionally supportive had more psychiatric problems and lower self-esteem. In addition intrusiveness of the parents was found to be associated with low self-esteem. In a six-month follow up study, it was reported that the decrease of BMI during follow-up was affected by the emotional response of the caregivers (7).

### Study Limitations

The limitations of our study were the low number of the clinically obese patients who had consulted our outpatient clinic and failure to evaluate psychopathology in the parents. Also lack of follow-up can be considered a limitation of this study. It is debatable if the high EE is a risk factor for exhibiting obesity and mental problems in adolescents or a result of them. More follow-up studies are necessary to enhance our understanding of this field. The strengths of our study were evaluating EE using a self-report scale specially designed for adolescents and comparing the obese subjects with their healthy peers. Additionally, this study is the first controlled study to evaluate the relationship between perceived EE and obesity in adolescents.

## Conclusion

To conclude, we can recommend that EE should be considered when planning treatment interventions to enhance compliance in obese adolescents.

## Figures and Tables

**Table 1:**
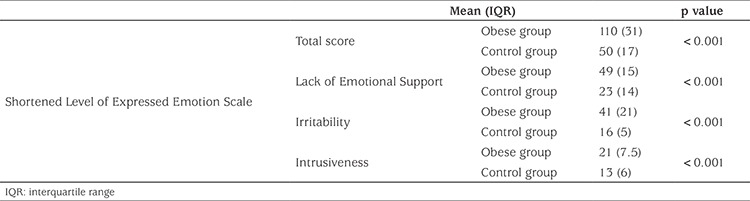
Perceived expressed emotion scores in the obese and control groups

**Table 2:**
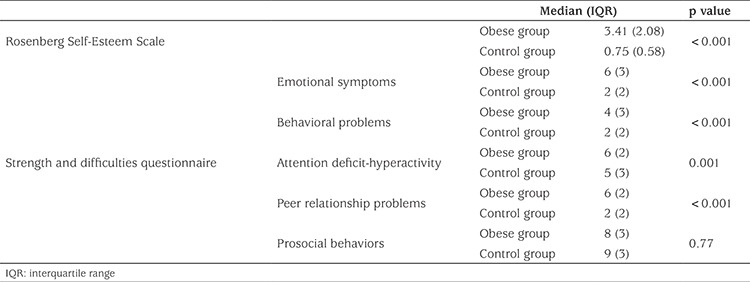
Rosenberg Self-Esteem scores and Strengths and Difficulties Questionnaire scores in the obese and the control groups

**Table 3:**
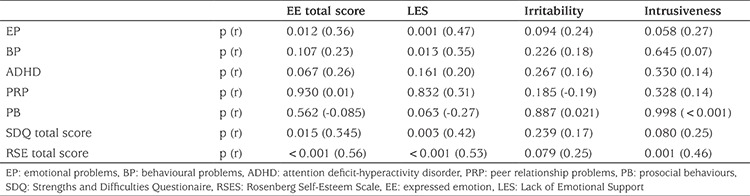
Correlation (Spearman rho) between expressed emotion and Rosenberg Self-Esteem or Strengths and Difficulties Questionnaire scores in the obese group
